# Quantification of lipid bodies in monocytes from patients with periodontitis

**DOI:** 10.1002/cre2.340

**Published:** 2020-11-14

**Authors:** Priscilla F. Naiff, Selma A. S. Kuckelhaus, Danilo Corazza, Luciana M. Leite, Shirley Couto, Mariangela S. deOliveira, Luander M. Santiago, Larissa F. Silva, Laudimar A. Oliveira, Daniela C. Grisi, Valeria M. A. Carneiro, Maria do C. M. Guimarães

**Affiliations:** ^1^ Periodontics Division Health Secretary of Amazon State and Health Secretary of Manaus Manaus Brazil; ^2^ Laboratory of Histological Techniques, Faculty of Medicine University of Brasilia Brasilia Brazil; ^3^ Laboratory of Cellular Immunology, Faculty of Medicine University of Brasilia Brasilia Brazil; ^4^ Faculty of Dentistry University of Brasilia Brasilia Brazil; ^5^ Endodontics Division University of Brasilia Brasilia Brazil; ^6^ Periodontics Division University of Brasilia Brasilia Brazil

**Keywords:** atherosclerosis, blood cells, lipid droplets, monocyte, oral health, periodontal diseases

## Abstract

**Objectives:**

For the first time in the history of periodontics, the production of lipid bodies by monocytes was assessed from blood of patients with periodontitis in comparison to systemically healthy individuals. The purpose of this study was to compare the lipid body frequency within monocytes between healthy patients and those with periodontal disease.

**Materials and Methods:**

A total of 30 participants (11 males and 19 females), were divided between orally healthy control subjects (C, *n* = 16) and periodontitis subjects (P, *n* = 14), in a cross‐sectional study. Both groups were systemically healthy. The following clinical periodontal parameters were assessed: probing depth, clinical attachment level, visible plaque index and gingival bleeding on probing index. Blood samples were collected to obtain monocytes containing lipid bodies, which were analyzed by light microscopy.

**Results:**

The periodontitis group demonstrated a higher corpuscular index than the control group (nonopsonized *p* = .0296 or opsonized *p* = .0459; Mann–Whitney). The frequency of monocyte cells containing lipid bodies (basal *p* = .0147, opsonized *p* = .0084 or nonopsonized, *p* = .026; Mann–Whitney) was also higher compared to those observed in healthy individuals.

**Conclusions:**

The data suggest that periodontitis may contribute to a higher production of lipid bodies. It was also hypothesized that a major production of lipid bodies by monocytes in severe periodontitis, compared to orally healthy subjects, could interfere with the innate immune response or represents a higher reservoir of cholesterol esters within macrophages and a major risk to systemic implications, such as atherosclerosis.

## 1 INTRODUCTION

Periodontitis is an infectious and inflammatory disease that affects both the teeth and their supporting tissues (Naiff, Carneiro, & Guimarães, 2018). The disease constitutes a major risk of dental mutilation, masticatory dysfunction, cardiovascular disease (Siddeshappa et al., 2016) and has been considered the sixth complication of diabetes mellitus (Löe, 1993; Naiff et al., 2018).

Periodontopathogens may stimulate cells from the innate immune system as monocytes and neutrophils, leading to an exacerbation of the local and systemic inflammatory response, depending on the host's health conditions (Naiff, Orlandi, & Dos‐Santos, 2012). These microorganisms and their toxins can disseminate intravascularly from the periodontal pockets throughout the human body (Naiff et al., 2018).

Most eukaryotic cells contain varying amounts of cytosolic lipid inclusions called lipid bodies (LBs) or lipid droplets, which regulate the hydrolysis and storage of neutral lipids (Vallochi, Teixeira, Oliveira, Maya‐Monteiro, & Bozza, 2018). Their structure comprises an external monolayer of phospholipids and a central nucleus rich in neutral lipids as triglycerides and sterol esters (Vallochi et al., 2018).

Intracellular changes related to the formation of LBs may occur within the cells in response to a variety of inflammatory factors. The formation of these lipid inclusions during infectious processes is a well‐regulated phenomenon that may have implications in the pathogenesis and an important role in modulating the immune response (Vallochi et al., 2018).

These corpuscles are characterized as dynamically and functionally active. Additionally, the intracellular accumulation of lipids in these organelles is associated with diseases of high public health relevance such as hepatitis (Miyanari et al., 2007) and dengue (Lima, 2015). However, no study in the current literature has evaluated the intracellular accumulation of LBs in cells from patients with periodontal diseases.

Considering the infectious and inflammatory nature of periodontal diseases, the purpose of this study was to analyze the impact of periodontitis on the immunological structural intracellular changes related to the accumulation of LBs in monocytes from the peripheral circulation, compared to healthy individuals.

## 2 MATERIAL AND METHODS

### 2.1 Study design

This study followed the Strengthening the Reporting of Observational Studies in Epidemiology (STROBE) guidelines. This cross‐sectional observational study was prospectively evaluated. Data collection was conducted for 1 year (from April 2016 to April 2017). Subjects were recruited at the Clinic of Periodontology of University Hospital of Brasília (HUB), Distrito Federal, Brazil. Thirty individuals (11 males and 19 females) underwent periodontal examination by a single examiner (PFN) and they were allocated into two groups: periodontitis (P) or controls (C).

### 2.2 Ethical considerations

This study was approved by the Human Research Ethics Committee of the University of Brasilia (UnB) (process number 46609515.7.0000.0030), in accordance with Brazilian legislation, which complies with the Declaration of Helsinki in 2013. All subjects were individually informed about the proposed study and agreed to participate by signing an informed consent form.

### 2.3 Sample size estimation

**FIGURE 1:**
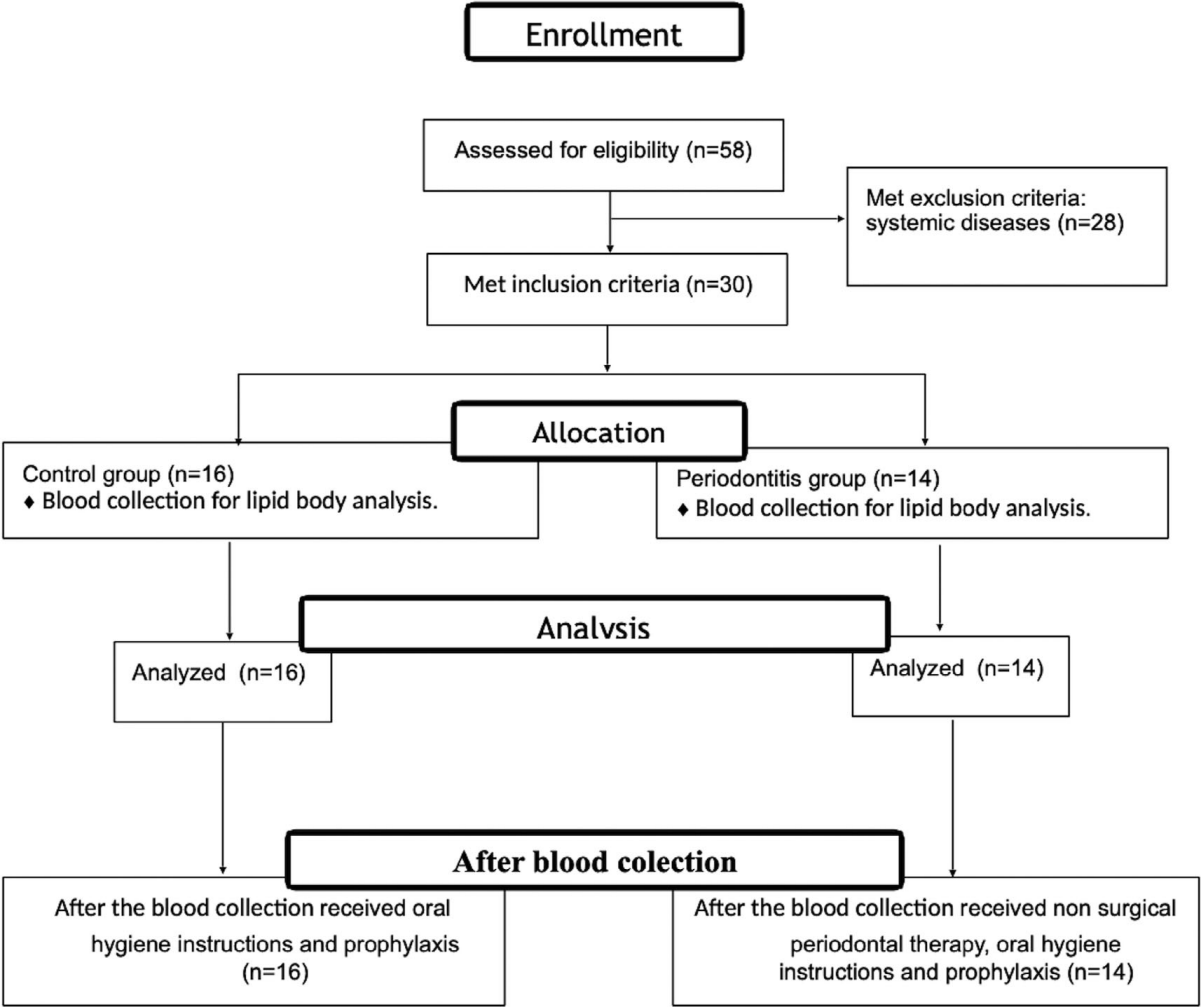
The flowchart of the study according to the Strengthening the Reporting of Observational Studies in Epidemiology (STROBE)

Sample size calculation was estimated based on an alpha error of 0.05 and a 95% confidence level. The semiannual average number of patients seen in the clinic of Periodontics at HUB (*n* = 30) was considered as the population size and the prevalence of periodontitis in the adult Brazilian population was estimated as 19.4%, according to the literature (Ministério da Saúde, 2012). The estimated sample size was 27, including patients and controls. It was decided to include all participants who agreed to volunteer and met the inclusion criteria. The final sample size was 30 (16 controls and 14 patients with periodontitis) (Figure 1).

### 2.4 Selection of participants and assessment of periodontitis

The inclusion criteria for participation in the study were patients with periodontitis (P) without any systemic disease, ≥30 years old and a minimum of 15 teeth. The diagnosis of periodontitis was defined according to the new classification of periodontal diseases and conditions (Papapanou et al., 2018) as follows: clinical and radiographic diagnosis of periodontitis at ≥2 non‐adjacent teeth (CAL ≥3 mm, PD ≥4 mm, confirmed by radiographic bone loss in the coronal third or extending to the middle or apical third of the root and beyond, ≥stage II; grade A). The control group (C) was composed by orally and systemically healthy individuals, ≥30 years old and a minimum of 20 teeth. The periodontal health criteria used for controls (Chapple et al., 2018) were the absence of interproximal or buccal clinical attachment loss (CAL) ≥3 mm, probing depth (PD) ≤3 mm, bleeding on probing (BOP) <10% and no radiographic evidence of bone loss.

The following conditions were considered exclusion criteria: smokers and nonsmokers who have smoked in the last 5 years, periodontal treatment and antimicrobial therapy for systemic conditions or topical oral use in the last 12 months, use of anti‐inflammatory, corticoid and immunosuppressant agents, pregnant or lactating women, autoimmune, infectious, allergic, renal and endocrine diseases, cancer, morbid obesity (body mass index [BMI] > 40 kg/m^2^) or malnutrition (BMI < 18.5 kg/m^2^) (National Institutes of Health, 1998).

Initially, all volunteers were interviewed in order to collect epidemiological data, including age, sex, and smoking habits. Individuals who had never smoked or had quit smoking more than 5 years before the interview were considered nonsmokers.

Clinical examination was performed in a full‐mouth model, with the exception of third molars, at six sites on each tooth. Probing depth (PD), clinical attachment level (CAL), visible plaque index (PI) (Ainamo & Bay, 1975) and gingival bleeding on probing (BOP) (Ainamo & Bay, 1975) were recorded with a periodontal probe (CP 15 screening probe).

All participants of the study received oral self‐hygiene instructions for correct interdental cleaning and teeth brushing. Subjects without periodontal diseases underwent dental prophylaxis. Patients with periodontitis underwent mechanical periodontal therapy.

### 2.5 Blood sampling for biochemical analysis

Fasting of 8 hr for blood collection and samples were collected and analyzed at Sabin Laboratory, Brasilia—Distrito Federal, Brazil to verify the lipid profile from the participants of the study.

### 2.6 Obtaining peripheral blood mononuclear cells

In order to obtain mononuclear cells from peripheral blood (PBMC), a previous protocol was used and adapted from Muniz‐Junqueira, dos Santos‐Neto, and Tosta (2001). 10 ml of venous blood were collected in a heparinized vacutainer tube. After centrifugation (2,000 rpm for 10 min, 4°C), supernatant serum was collected, aliquoted and frozen at −80°C. The precipitate composed of red blood cells, leukocytes and platelets was resuspended with phosphate‐buffered saline (PBS) at approximately 4°C, pH 7.2, in the same volume of removed serum. Then, the resuspended content was gently placed on the percoll (density 1,077, 3 ml per 5 ml of blood) followed by centrifugation, 3,000 rpm for 15 min at 4°C. The layer of mononuclear cells was then transferred to a new tube. For removal of the percoll residues, the content was resuspended in 10 ml of PBS at approximately 4°C, pH 7.2, and centrifuged at 2,000 rpm for 10 min at 4°C. The supernatant was discarded; the pellet resuspended in 10 ml of chilled PBS and centrifuged 1,000 rpm for 10 min at 4°C for platelets removal. The supernatant was discarded, and the pellet resuspended in 2 ml of Roswell Park Memorial Institute (RPMI) incomplete medium, pH 7.2 and kept in an icebox until the cell count was complete.

For the mononuclear cell counting obtained after the homogenization of the solution with Pasteur pipette, 20 μl were removed and diluted with 80 μl of 0.05% nigrosin in PBS. The cells were counted in a Neubauer's chamber by light microscopy. The number of live, dead and total number of cells were obtained.

### 2.7 Quantification of lipid bodies

The quantification of LBs was performed by the red oil cytochemical labeling technique (Oil red—O), which is a dye with great solubility for lipophilic substances, and which is presented in red color in monocyte cytoplasm. This protocol was adapted from the association of two techniques: Muniz‐Junqueira, Peçanha, Silva‐Filho, Cardoso, and Tosta (2003) and Borges et al. (2017).

PBMC were resuspended in RPMI (10^6^ PBMC per 500 μl) and the solution was distributed in 24 excavations culture plates with circular sterile glass laminates previously inserted in each well. The plate was incubated for 2 hr in a humid chamber with 5% CO_2_ at 37°C for adhesion of monocytes to the laminules and subsequently washed twice with sterile fetal bovine serum (FBS) at 37°C, for basal and nonsensitized samples. Sensitized samples were washed with human serum from own subjects instead of FBS. After wash, yeast (*Saccharomyces cerevisiae*) in the proportion of five yeasts to one monocyte (5:1) was added in complete RPMI medium and incubated for 30 min in sensitized and nonsensitized samples. Yeast was not added to basal samples. A new wash with FBS at 37°C was performed.

The slides with basal samples had no stimulus and no yeast. Slides with sensitized samples had yeast and opsonin from the donor of the sample itself, while the slides with nonsensitized samples had yeast but did not have opsonin.

The cells were fixed with 4% paraformaldehyde for 30 min and then washed twice with PBS, pH 7.2, and once with 60% isopropyl alcohol. Monocytes were stained for 15 min with a red oil solution. Oil red was filtered three times in a 0.22 μm filter and prepared at the time of use from a 0.5% stock solution. The excess of dye was removed, and the excavations were washed once with 1 ml of 60% isopropyl alcohol and then twice with 1 ml of distilled water. The cell nuclei were stained with Mayer's hematoxylin for 5 min, washed again with distilled water and the laminules were mounted on glass slides containing a thin layer of gelatinous medium (10 g of gelatin, 60 ml of distilled water, 70 ml of glycerol and 0.25 g of phenol).

By this method, lipid droplets appear as circular structures that redden in the cytoplasm of monocytes (Figure S1). The percentage of monocytes presenting LBs in the cytoplasm (MØ%) was determined, and the average number of LBs within monocytes (MCL) was counted. Then, the corpuscular index (CI) was established by multiplying MCL and MØ%. For each patient, 200 monocytes basal, sensitized and nonsensitized were blinded evaluated by a single examiner (MO).

### 2.8 Statistical analysis

Statistical analysis was performed using Prism® software (GraphPad, San Diego, CA). Variables in the samples were previously verified for normality by the Kolmogorov–Smirnov test. To verify the differences between groups, an unpaired *t* test or Mann–Whitney test, was performed for variables with Gaussian or non‐Gaussian distribution, respectively. The level of significance was set at *p* < .05.

## 3 RESULTS

### 3.1 Demographic data, clinical and laboratory parameters

Each subject who volunteered to participate in the study and met the inclusion/exclusion criteria was included in the study. Thirty (30) participants, 11 (36.7%) males and 19 (63.3%) females were enrolled at the study.

Two groups were assessed as follows: (a) Periodontitis group (P) consisted of 14 patients (8F; 6M), age 45 ± 9 and mean number of teeth 23 ± 6; and (b) Control group (C) consisted of 16 healthy volunteers (11F; 5M), age 42 ± 9 and mean number of teeth 27 ± 2.

Regarding clinical parameters, patients with periodontitis had a significantly higher PI, BOP, CAL and PD compared to controls (Table 1).

**TABLE 1:** Clinical periodontal data and body mass index from the participants of the study

	Control group (*n* = 16)	Periodontitis group (*n* = 14)	*p* value
PI (%)	12.1 ± 2.9	79 ± 27.4	<.0001
BOP (%)	7.5 ± 1.3	48 ± 26.1	<.0001
CAL^a^ (mm)	2.8 ± 0.2	6.3 ± 1.9	<.0001
PD^a^ (mm)	2.1 ± 0.4	6.1 ± 1.7	<.0001
Extension (%)	0	51 ± 29.8	<.0001
BMI (kg/m^2^)	25.6 ± 2.9	27.7 ± 5.7	.2

*Notes:* Unpaired *t* test. Values are expressed as mean ± standard deviation; *p* value significant when <.05.

Abbreviations: BMI, body mass index; BOP, gingival bleeding on probing; CAL, clinical attachment loss; Extension, extension of periodontitis (<30%, localized periodontitis, ≥30%, generalized periodontitis); PD, probing depth; PI, plaque index.

^a^ Deeper PD or major CAL per tooth.

All values obtained for the lipid profile were within the reference values for normality in both groups (Table 2).

**TABLE 2:** Lipid profile from the participants of the study

	Control group (*n* = 16)	Periodontitis group (*n* = 14)	*p* value	RV
TC (mg/dl)	172.5	189.5	>.05	<190
	(161.3–219.3)	(176.8–212)		
HDL (mg/dl)	52	51	>.05	>40
	(44.5–56.75)	(45.5–61.5)		
LDL (mg/dl)	102.5	109.5	>.05	<130
	(84.5–142)	(102.3–123.5)		
TRGL (mg/dl)	108	121	>.05	<150
	(77–173.5)	(69.75–147)		

*Notes:* Mann–Whitney test. Values are expressed as median/interquartile range.

Abbreviations: HDL, high density lipoprotein; LDL, low density lipoprotein; TC, total cholesterol; TRGL, triglycerides; RV, reference values.

### 3.2 Lipid bodies

Patients with periodontitis showed a significantly higher corpuscular index (CI) for both nonsensitized (CI = 13.5; *p* = .0296, Mann–Whitney) and sensitized samples (CI = 26; *p* = .0459, Mann–Whitney) than controls (CI = 4.2 and 7.5, respectively), differently from basal samples that did not show a significant difference (*p* = .18).

**FIGURE 2:**
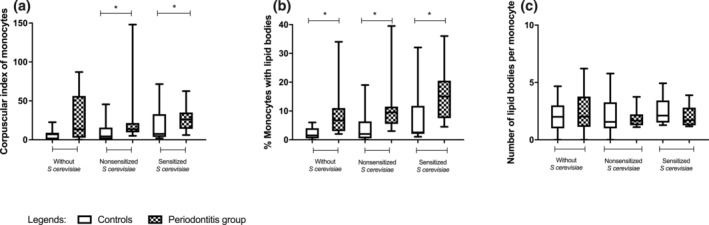
Production of lipid bodies by peripheral blood monocytes evaluated by the corpuscular index (% of monocytes with lipid bodies × mean number of lipid bodies per monocyte). Values are shown as medians, quartiles, maximum and minimum values. *p < .05, Mann–Whitney

The percentage of monocytes presenting LBs in the cytoplasm (MØ%) was higher in the periodontitis group (6.75 basal, *p* = .0147; 9.5 nonsensitized, *p* = .026 and 15 sensitized, *p* = .0084) compared to the control group (1.5, 2 and 2.5, respectively). There was no difference in the average number of LBs per monocyte (MBL) between the studied groups (*p* > .05). Figure 2 shows the quantification of LBs per group according to MØ%, MBL and CI.

The complete information on the quantification of LBs in the current research was presented in Table S1.

## 4 DISCUSSION

This research is the first step to a deeper understanding about the effect of periodontitis on the production of LBs by peripheral monocytes.

LBs are organelles commonly found scattered in the cytoplasm of healthy human cells, but some pathological conditions such as neoplasia, atherosclerosis, bacterial sepsis, acute respiratory distress syndrome, arthritis and mycobacterial infections may be associated with their increased number (Vallochi et al., 2018). LBs are the main organelles producing cellular eicosanoids and the key in the intracellular signaling and inflammatory process (Vallochi et al., 2018).

Eicosanoids (prostaglandins, leukotrienes and others) are produced from the transformation of arachidonic acid in response to inflammatory cytokines, such as tumor necrosis factor α (TNF‐α), interleukin 1β (IL‐1β) and lymphotoxin (Vallochi et al., 2018). They mediate cellular processes such as proliferation, apoptosis, metabolism and cellular migration (Vallochi et al., 2018). Besides eicosanoids, another group of inflammatory molecules was found within LBs: cytokines as IL‐16, chemokine ligand 5 (CCL5), among others (Bozza, Magalhães, & Weler, 2009).

LBs exist in several types of inflammatory cells besides noninflammatory ones, but some studies have shown that LBs in leukocytes, especially macrophages, increase during inflammation (Bozza et al., 2009; D'avila, Maya‐Monteiro, & Bozza, 2008; Vallochi et al., 2018) suggesting that LBs can function as large production sites for prostaglandins and other lipid mediators (Bozza et al., 2009; Vallochi et al., 2018).

The profile of patients in our study mainly involved individuals with stage III and grade A periodontitis, with generalized extension of the disease (reaching more than 30% of teeth present in the oral cavity). This profile corresponds to generalized severe periodontitis, which involves multiple teeth in a dentition which may lead to a partial or complete edentulism.

The total surface area of this periodontal inflammatory field is estimated to be the palm size. Immediate medical intervention should be done if a lesion of this size was on the skin. However, periodontitis is frequently ignored by health professionals, even though it may be associated with a large number of diseases and systemic conditions (Mohangi, Singh‐Rambirich, & Volchansky, 2013; Naiff et al., 2018).

Lipid profile from all participants was within the reference values for normality. This is important to avoid *bias* in the study protocol.

Body mass index did not differ between groups. According to the World Health Organization, most of the study's participants were already overweight (National Institutes of Health, 1998). It is worth mentioning that BMI between 20 and 25 kg/m^2^ provides a better survival rate and that the relative risk of mortality increases in individuals outside this range (Di Angelantonio et al., 2016).

Basal levels of the total amount of inactivated monocytes LBs, without any stimulus, showed no difference between groups.

However, a higher corpuscular index was found in patients with periodontitis after phagocytosis of nonopsonized and opsonized *S. cerevisiae* than in controls. This is probably due to a higher frequency of monocytes producing LBs, since there was no difference in the average number of LBs present in each cell, between these populations. This leads us to assume that when an antigen incites the immune system in individuals with periodontitis, it may stimulate a higher number of monocytes to produce LBs than in oral healthy individuals. This usually occurs by both opsonins and cell surface receptors. However, although a large number of cells containing LBs in their cytoplasm were observed, there was no cell overfunction, that is, the production of LBs by monocytes remained unchanged.

Considering the inflammatory and infectious nature of periodontal diseases, our findings agree with other studies, which report a direct association between parasites (Chagas's disease, malaria) (Vallochi et al., 2018) or infectious diseases (hepatitis C, tuberculosis) (Miyanari et al., 2007; Vallochi et al., 2018), and the increased number of LBs. This gives us the idea that LBs could be associated with the etiopathogeny of periodontitis.

From another point of view, due to the higher frequency of monocytes with LBs found in the blood circulation of patients with periodontitis than in controls, the disease, through different mechanisms not completely elucidated, may cause a greater stimulation of the immune system and this can harm systemic health because LBs are fundamental parts in the inflammatory process (Bozza et al., 2009; D'avila et al., 2008; Vallochi et al., 2018).

Periodontitis may cause bacteremia and enhance atherosclerotic plaque formation since some microorganisms related to periodontal diseases as *Porphyromonas gingivalis* were detected in atherosclerotic plaques (Schenkein, Papapanou, Genco, & Sanz, 2020). *P. gingivalis* or their products can also interact with platelets (directly or through the vascular endothelium) and promote prothrombotic effects (Schenkein et al., 2020). However, other oral pathogens as *Streptococcus mutans* were also found in atheromatous plaque samples (Nakano et al., 2006). Thus, it seems that the disruption of epithelial integrity of periodontal pockets may also provide an entry point for nonperiodontal pathogens, such as those normally found in teeth affected by caries.

Proinflammatory cytokines, which have been reported to be associated with periodontitis, are also involved in atherothrombogenesis (Ridker & Silvertown, 2008). In addition, bacterial products (LPS, external membrane vesicles, or fimbriae), cytokines, and chemokines resulted from the infectious and inflammatory periodontal process fall into the bloodstream and may stimulate overregulation of the surface receptors of endothelial cell, in addition to the expression of adhesion on vascular endothelium (Kebschull, Demmer, & Papapanou, 2010). From this point of view, periodontitis can be a risk factor for CVD as atherosclerosis.

This occurs through endothelial activation that promotes chemotaxis for circulating monocytes. These cells adhere to the activated endothelium (Kebschull et al., 2010). Due to molecular mimicry, immunoglobulins against specific bacterial proteins act as autoantibodies and induce apoptosis in the endothelium. Monocytes then migrate to the subendothelial space and differentiate into macrophages. There, they pick up low‐density oxidized lipoprotein (LDL) and become foam cells (Kebschull et al., 2010).

At this moment, LBs containing an accumulation of cholesterol esters in foam cells could also be an important mechanism in the development of atherosclerosis, but little is known about the protein compositions of LBs in foam cells and how the formation and mobilization of LBs are regulated in the context of atherosclerotic plaque formation (Goldberg et al., 2018).

Foam cells suffer apoptosis and result in the accumulation of lipids in the subendothelial space, contributing to the formation of atheromatous plaques. Besides, invading periodontal pathogens induce the proliferation of smooth muscle cells in the formation of the intima and neointima (Kebschull et al., 2010).

The development of extracellular matrix and the extravasation of T lymphocytes result in the formation of a fibrous envelope covering the atheroma. The fibrous cap and its prothrombotic components are exposed after apoptosis of the endothelial cells (Kebschull et al., 2010). The enzymatic degradation of the extracellular matrix results in the rupture of the plaque with the consequent exposure of its prothrombotic components and thrombus formation, leading to occlusion of the vessels. Clinically, this manifests as acute myocardial infarction in the case of an occluded coronary artery, or a stroke in the case of an occluded cerebral vessel (Kebschull et al., 2010).

Thus, if periodontitis in humans is capable of promoting a systemic increase in the number of macrophages containing LBs, including the vascular endothelium, severe periodontal disease may be an important risk factor for the formation of an atherosclerotic plaque.

A possible limitation of this study is the extrapolation of an in vitro laboratory finding for a clinical condition involving a multiplicity of interrelated functional factors. Therefore, our assumptions can be confirmed through further investigation.

Another limitation that may be considered in this study is the sample size. Our sample was calculated based on the semiannual number of individuals seen in the clinic of Periodontics at HUB, a school hospital inside University of Brasília, Brazil. We intended to include more patients than was estimated after sample size calculation, but we could not reach a very expressive number of patients after using a rigorous inclusion and exclusion criteria. Most of the individuals interviewed had systemic diseases such as diabetes, were smokers or had partial edentulism (with fewer teeth than the minimum required for each group) which could compromise the association between periodontitis with changes in individual immune responses and create *bias* in the study. Another complicating factor was that there was a low demand for dental care by the population affected by periodontitis, perhaps since the disease does not often have painful associated symptomatology. Patients often only seek specialized dental care when their teeth already have evident mobility with the indication of multiple exodontia.

Another possible limitation of this work was that we used dead *S. cerevisiae* and not periodontal pathogens to investigate the production of LBs by monocytes. When using live bacteria, their virulence factors may influence the activation of cells for LBs production. Thus, when investigating this process, two lines of reasoning can be defined: The production of LBs can be analyzed directly at the site of infection/inflammation in the periodontium or, systemically. However, as our aim was to evaluate the effects of this interaction on the host in monocytes from blood and not from periodontitis sites, this justifies another stimulus provided to the cell, including yeast. Zymosan particles have been used as a model for the recognition of microbes by the innate immune system for over 50 years (Underhill, 2003) (in fact, almost 70 years). Furthermore, *S. cerevisiae* was chosen as the particle to be phagocytosed because it is taken up by the same receptors in monocyte/macrophages as those that phagocytose pathogenic bacteria present in periodontitis (Underhill & Ozinsky, 2002).

Despite these limitations, our findings reinforce the concept that periodontitis may promote significant immunological changes in human peripheral blood cells whose systemic impacts need to be better understood.

## 5 CONCLUSIONS

The present study supports the argument that periodontitis may stimulate a higher quantity of peripheral blood monocytes to produce LBs when activated by antigens, systemically. The higher number of LBs found in monocytes from periodontitis patients than in healthy individuals could lead to systemic inflammation and predispose patients to systemic diseases, such as atherosclerosis. Nonetheless, considering the in vitro setting of this study, the interpretation of the findings about the stimulation of the immune system should be made cautiously.

## CONFLICT OF INTEREST

The authors declare that there is no conflict of interest in this study.

## AUTHOR CONTRIBUTIONS

Priscilla F. Naiff contributed to conception, design, performed laboratory experiments, data acquisition and interpretation, performed statistical analyses, prepared and drafted the manuscript. Selma A. S. Kuckelhaus contributed to conception, design, performed statistical analyses, drafted and critically revised the manuscript. Danilo Corazza, Luciana M. Leite, and Shirley Couto performed laboratory experiments, and critically revised the manuscript. Mariângela Oliveira performed laboratory experiments, data acquisition and analysis, and critically revised the manuscript. Luander M. Santiago and Larissa F. Silva contributed to data collection and performed laboratory experiments, and critically revised the manuscript. Laudimar A. Oliveira, Daniela C. Grisi, Valeria M. A. Carneiro, and Maria do C. M. Guimarães contributed to conception, design, and critically revised the manuscript. All authors gave their final approval and agree to be accountable for all aspects of the work.

## Supporting information


**Figure S1**: Monocytes from a periodontitis patient presenting lipid bodies in their cytoplasm (light microscopy).
**Table S1**: Quantification of lipid bodies in the cytoplasm of monocytes.

## Data Availability

The data that supports the findings of this study are available in the supplementary material of this article and other information about the study can be obtained from the corresponding author upon reasonable request.
