# Supplementary material for: Quantification of lipid bodies in monocytes from patients with periodontitis
Source: Clin Exp Dent Res. 2020 Nov 14;7(1):93–100. doi: 10.1002/cre2.340 (PMC7853883; doi:10.1002/cre2.340)
Supplement: Supplementary file 1 — Figure S1: Monocytes from a periodontitis patient presenting lipid bodies in their cytoplasm (light microscopy). Table S1: Quantification of lipid bodies in the cytoplasm of monocytes. [file CRE2-7-93-s001.doc]

Appendix Figure 1


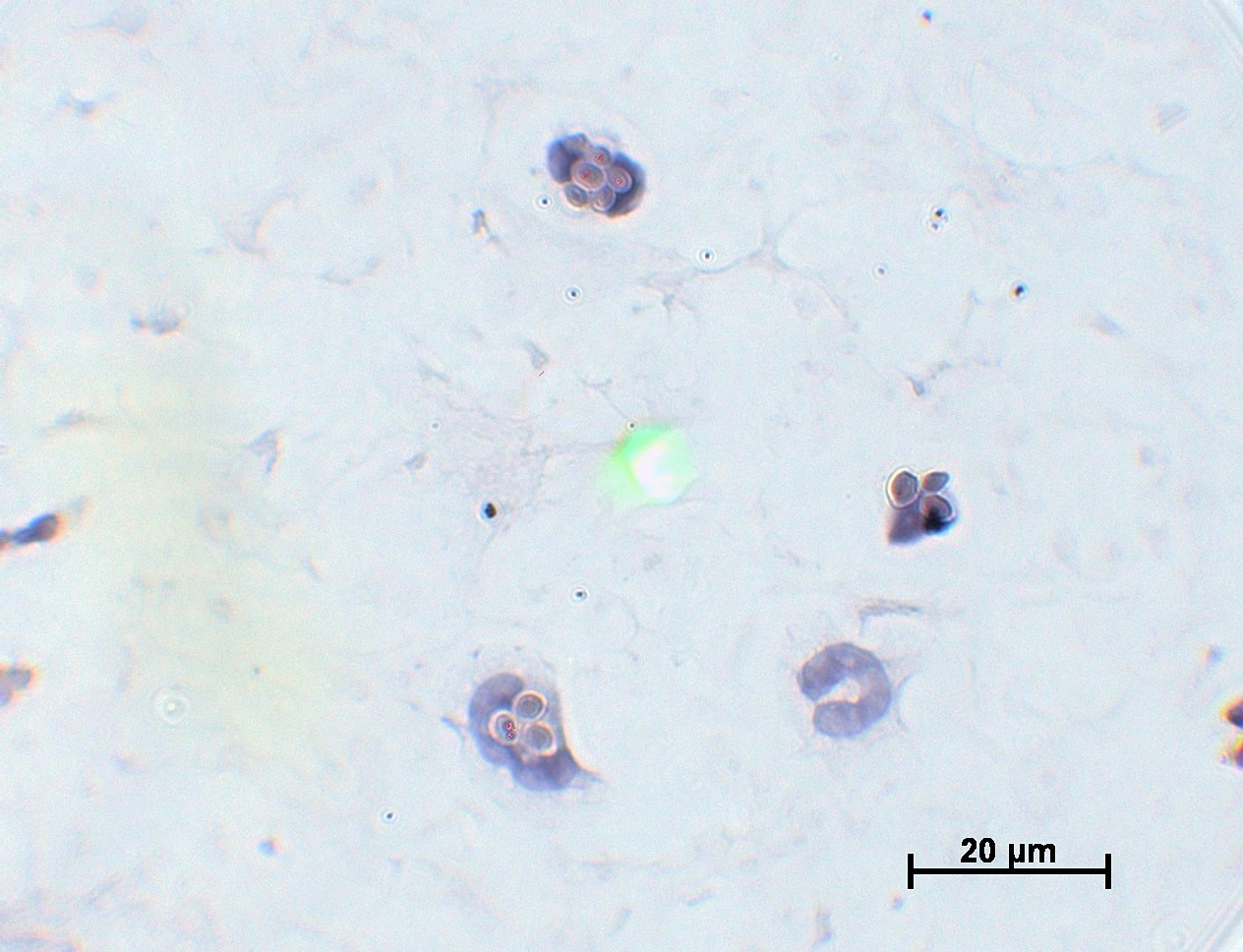


Figure 1A. Monocytes from a periodontitis patient presenting lipid bodies in their cytoplasm (Light microscopy).

Appendix Table 1:

**Table 1A**. Quantification of lipid bodies in the cytoplasm of monocytes.

| **BASAL** | **C** | **P** |
| --- | --- | --- |
|  | *Median (interquartile range)* | |
| CI | 7^1^  (0.5 – 9) | 13.25  (2.6 – 56.2) |
| MØ% | 1.5^1*^  (0.5 – 4) | 6.75  (3 – 11) |
| MBL | 2^2^  (1 – 3) | 2.02  (1.12 – 3.7) |
| **NONSENSITIZED** |  | |
| CI^*^ | 4.2^1*^  (0.62 – 15.6) | 13.5  (9.5 – 21.5) |
| MØ%^*^ | ^21*^  (0.5 – 6.4) | 9.5  (5.5 – 11.5) |
| MBL | 1.5^1^  (1 – 3.2) | 1.6  (1.2 – 2.2) |
| **SENSITIZED** |  | |
| CI^*^ | 7.5^1*^  (3.6 – 32.8) | 26  (14 – 35) |
| MØ%^*^ | 2.5^1*^  (2 – 11.7) | 15  (7.5 – 20.5) |
| MBL | 2.1^1^  (1.5 – 3,4) | 1.7  (1.26 – 2.8) |

^1^Mann-Whitney test and ^2^Unpaired T-test. Values are expressed as median (interquartile range). ^*^*p*-value < 0.05 (C x P) . Legends: C = control group; P = periodontitis group; CI = corpuscular index; MØ% = monocytes presenting lipid bodies in the cytoplasm; MBL = mean number of lipid bodies per monocyte.

**QUANTIFICATION OF LIPID BODIES IN MONOCYTES FROM PERIODONTITIS PATIENTS.**

PRISCILLA F. NAIFF*; SELMA A.S. KUCKELHAUS†; DANILO CORAZZA ‡; LUCIANA M. LEITE ‡; SHIRLEY COUTO ‡; MARIÂNGELA OLIVEIRA‡; LUANDER M SANTIAGO§; LARISSA F. SILVA§; LAUDIMAR A. OLIVEIRA ‖; DANIELA C. GRISI¶; VALERIA M.A. CARNEIRO¶; MARIA DO CARMO M. GUIMARÃES ¶
